# Meta-Analysis of Studies Incorporating the Interests of Young Children with Autism Spectrum Disorders into Early Intervention Practices

**DOI:** 10.1155/2012/462531

**Published:** 2012-05-14

**Authors:** Carl J. Dunst, Carol M. Trivette, Deborah W. Hamby

**Affiliations:** ^1^Orelena Hawks Puckett Institute, 8 Elk Mountain Road, Asheville, NC 28804, USA; ^2^Orelena Hawks Puckett Institute, 128 South Sterling Street, Morganton, NC 28655, USA

## Abstract

Incorporating the interests and preferences of young children with autism spectrum disorders into interventions to promote prosocial behavior and decrease behavior excesses has emerged as a promising practice for addressing the core features of autism. The efficacy of interest-based early intervention practices was examined in a meta-analysis of 24 studies including 78 children 2 to 6 years of age diagnosed with autism spectrum disorders. Effect size analyses of intervention versus nonintervention conditions and high-interest versus low-interest contrasts indicated that interest-based intervention practices were effective in terms of increasing prosocial and decreasing aberrant child behavior. Additionally, interest-based interventions that focused on two of the three core features of autism spectrum disorders (poor communication, poor interpersonal relationships) were found most effective in influencing child outcomes. Implications for very early intervention are discussed in terms addressing the behavior markers of autism spectrum disorders before they become firmly established.

## 1. Introduction

Children with autism spectrum disorders often manifest difficulties with communication and interpersonal relationships as well as manifest obsessive and repetitive behaviors [1, 2]. The latter are generally described as preoccupations, restricted and repetitive behavior, compulsions, stereotypes, and limited interests [3–5]. The terminology most often used to describe the limited interests of individuals with autism spectrum disorders includes narrow interests [6], ritualistic interests [7], circumscribed interests [8], and perseverative interests [9]. As stated into the *Diagnostic and Statistical Manual of Mental Disorders-IV* [10], markedly restricted, repetitive, and stereotyped patterns of interests, behavior, and activities are one of the criteria for diagnosing autism spectrum disorders.

The *Diagnostic and Statistical Manual of Mental Disorders-IV-Text Revision *[11] includes additional information about the patterns of behavior associated with the restricted and repetitive interests, behavior, and activities of individuals with autism spectrum disorders. These include the preoccupation with one or more stereotyped and restricted patterns of interest that is abnormal either in intensity or focus; inflexible engagement in specific, nonfunctional routines or rituals, stereotyped and repetitive motor mannerisms; persistent preoccupation with parts of objects. 

The ways in which limited interests have been incorporated in studies of individuals with autism spectrum disorders have varied considerably, and as of yet, no attempt has been to determine if different approaches have different results or consequences. Additionally, close inspection of how the interests of individuals with autism spectrum disorders are described or defined indicates that investigators rarely differentiate between different types of interests making it difficult to discern whether the various ways of defining and operationalizing child interests matter in terms of explaining child outcomes and benefits. This state of affairs was addressed in the meta-analysis described in this paper by operationally defining two types of interests and investigating the manner in which either or both types were related to differences in child outcomes.

The main purpose of the meta-analysis was to determine the differential effectiveness of interest-based interventions with young children with autism spectrum disorders 2 to 6 years of age. One goal was to integrate available evidence to determine if interest-based practices are warranted as an intervention for young children with autism spectrum disorders. Meta-analyses are especially suited for achieving this goal because they permit a determination of the overall or common effect of interventions designed to have the same or similar effects [12]. A second goal was to identify the conditions under which interest-based interventions have similar or dissimilar effects [13]. Meta-analyses are also well suited for achieving this goal because pooling results across studies permits evaluation of the differential effects of interventions examined in different ways [14]. A third goal was to identify gaps in knowledge in order to inform future research to be able to better understand the characteristics of interest-based intervention practices that are most effective in terms of influencing the behavior of young children with autism spectrum disorders. 

The studies in the meta-analyses included only children 6 years of age and younger since recent advances in the early assessment of autism spectrum disorders make it possible to identify the behavior markers of the disorder long before the markers become firmly established [15, 16]. This in turn makes it possible to intervene early in the children's lives to promote prosocial and lesson behavior excesses [17, 18]. We focused on studies of children younger than 6 years of age because no research synthesis or meta-analysis of interest-based studies with young children with autism spectrum disorders has yet to be conducted. The findings were expected to add to the knowledge base in terms of the characteristics of effective intervention practices designed to positively influence the learning, behavior, and development of children with autism spectrum disorders [19, 20], and especially in terms of research that has focused on the motivational features of intervention practices with children with autism (e.g., [17, 21–23]). 

### 1.1. Definition of Interests

Renninger et al. [24] as well as others [25] differentiate between two types of interests: personal and situational. Personal interests refer to those person characteristics that engage individuals in preferred or enjoyable activities [26]. Young children, for example, demonstrate personal interests in terms of preferences for certain objects, activities, and actions; prolonged attention to and engagement with people, objects, and events; positive social-affective behavior (e.g., smiling and laughing) while engaged in preferred activity; by choosing to interact or play with particular people or objects. Situational interests refer to interestingness of people, objects, activities, and so forth that evoke and sustain attention to and engagement with the social and nonsocial environment [27]. The situational interests of young children include, but are not limited to, sights and sounds that evoke attention; the characteristics and features of objects, materials, or toys that invite engagement; children's initiations in response to salient events; responses to violations of expectations.

Research with young children without autism spectrum disorders or other developmental disabilities shows that infants, toddlers, and older preschoolers engage in personally interesting activity [28, 29] and that they find many aspects of their social and nonsocial environments situationally interesting [30, 31]. Research also shows that young children with developmental disabilities exhibit both personal and situational interests [19, 32–34] and that children's interests function as a development-instigating characteristic influencing both behavioral and developmental outcomes [35]. Research syntheses and literature reviews of studies of the interests of young children with and without developmental disabilities show that variations in children's interests are related to variations in child behavior functioning and developmental outcomes [19, 36, 37]. In the largest majority of these studies, interest-based child participation in learning activities was associated with more positive and less negative child behavior and better developmental outcomes. The results, taken together, provide support for Bronfenbrenner's [38] contention that personal interests as well as situationally interesting aspects of the social and nonsocial environment function as development-instigating and development-enhancing factors influencing child behavior and learning.

### 1.2. Interest-Based Interventions

There are a number of different empirically validated interventions for treating the core features of autism [39–42]. These include, but are not limited to, behavioral and psychosocial interventions that target improvements in the communications and social interaction skills of young children with autism spectrum disorders. The particular kinds of interventions found most effective, for example, include pivotal response training [43], incidental and responsive teaching [44], interventions targeting improvements in joint attention [45], parent-mediated interventions [46], and behavioral interventions targeting decreases in problem behavior [47].

A novel and promising practice that is emerging as an alternative or supplement to other types of interventions is incorporating the interests of young children with autism spectrum disorders into early intervention practices to decrease aberrant and promote prosocial behavior [48, 49]. In one of the first demonstrations of an interest-based intervention with children with autism, Koegel et al. [50] found that engaging 4-to-13-year-old children with autism in child-preferred activities (personal interest) resulted in a discernable decrease in social avoidance behavior. In a study by Martin and Farnum [51] of 3-to-16-year-old children with autism spectrum disorders, introducing novel, unfamiliar dogs (situational interest) into the children's intervention sessions resulted in more prosocial and less stereotypic behavior compared to the use of noninterest-based objects. Similar results have been reported in other studies including children both younger and older than 6 years of age with autism spectrum disorders [52, 53].

## 2. Materials and Methods

### 2.1. Search Strategy

Studies were located using *autism* or autist* or “*autism spectrum disorder*” or “*rett syndrome*” or asperger* or “*asperger syndrome*” AND *interest* or excit* or motivate* or entertain* or *preference* or *preferred* or *favorite *or *choice *or “choice-mak* or “pref*object” or “*preferred object*” or preferred-object* AND *treatment* or *therapy *or *intervention* or “inter*therapy” or treat*therapy AND *infant* or *infancy *or *toddler *or preschool* as search terms. Both controlled vocabulary and natural language searches were conducted [54]. The search sources included PSYCHINFO, ERIC, MEDLINE, CINAHL, Academic Search Premier, Education Research Complete, and Rehabdata. These were supplemented by Google Scholar, Scirus, and Ingenta searches as well as a search of an extensive EndNote Library maintained by our institute. Hand searches of the reference sections of all retrieved journal articles, book chapters, books, dissertations, and unpublished papers were also examined to locate additional studies. 

Studies were included if the children in group design studies were all 6 years of age or younger; separate data were presented on individual children 6 years of age or younger in single participant design studies; the studies evaluated the effects of interest-based interventions on child behavior outcomes; Cohen's *d* effect sizes for either the baseline versus intervention or comparative conditions (e.g., preferred versus nonpreferred objects) could be computed from data in the research reports. Studies were excluded if they included children older than 6 years of age (e.g., [55, 56]), included children younger and older than 6 years of age but the data for the younger children were not reported separately (e.g., [50, 52]), or children's interests were important features of an intervention but variations in interests were not related to variations in the study outcomes (e.g., [57, 58]).

### 2.2. Search Results

Twenty-four intervention studies were located that included 78 children diagnosed with autism, pervasive developmental disorders, or autism spectrum disorders [59–84]. No intervention studies were located for children less than 6 years of age with either *Rett Syndrome* or *Asperger Syndrome*. The largest majority of the investigators (*N* = 16) reported using either or both the *Diagnostic and Statistical Manual of Mental Disorders* [10, 85] and the *Childhood Autism Rating Scale* [86, 87] for child diagnosis. The other investigators used different scales for child diagnosis [88–90]. 

The sample sizes in the studies ranged between 1 and 17 (median = 3). The mean child age in the studies was 52 months (range = 23  to  72). The mean developmental age of the children was 33 months (range = 10  to  67). Sixty-five children were male (83%) and 13 children were female (17%). Severity of the children's disorders was reported in 10 studies and estimated based on information included in 14 research reports. Severity was estimated based on behavioral and developmental information included in the research reports (e.g., a child within a normal range of intelligence who communicated verbally was classified as mild). The children were diagnosed with mild (*N* = 17), moderate (*N* = 21), severe (*N* = 6), mild-to-moderate (*N* = 17), and mild-to-severe (*N* = 17) autism spectrum disorders.

### 2.3. Research Designs

Sixteen studies used single-participant designs and eight studies used either between-conditions or between-group designs. The single-participant studies used multiple baseline designs across children (*N* = 6) or tasks (*N* = 1), ABAB designs (*N* = 5), or some other type of AB or ABA designs (*N* = 4). The eight between-condition and between-group designs all included some type of low- versus high-interest comparisons (e.g., choice versus no choice; preferred versus nonpreferred objects).

### 2.4. Interest Measures

The interest measures used by the investigators were described as narrow, ritualistic, obsessive, circumscribed, perseverative, personal, or situational interests. Interests were also described and measured in terms of child preferences (e.g., preferred versus nonpreferred objects) or child choices (e.g., choice versus no choice). Child interests were determined through observations (*N* = 11  studies), preference or choice assessments (*N* = 10  studies), or a combination of caregiver (parent or teacher) interviews and observations (*N* = 3  studies). The focus of investigation in all the studies was the consequences of incorporating the children's interests into the interventions albeit in different ways.

The definitions of personal and situational interests described in Section 1 were used to code the type of child interest used in each study. Studies were coded as using personal interests if a child interest assessment was conducted prior to the interventions and the children's preferences, likes, desires, and so forth were incorporated into the interventions to affect changes in child outcomes. Studies were coded as using situational interests if novel or highly salient materials were incorporated into the interventions to affect changes in child outcomes. Personal interests were used in 13 studies and situational interests were used in 11 studies.

### 2.5. Child Outcome Measures

The studies included a mix of negative or aberrant child behavior outcome measures and/or positive or prosocial child behavior outcome measures. The negative and aberrant child outcome measures included negative child affect, obsessional activity, problem and disruptive behavior, refusals, nonengagement, and self-stimulation. The positive and prosocial child outcome measures included positive child affect, appropriate social interactions and play, social approach, turn taking, joint attention, social and nonsocial engagement, language development, and task completion. The different types of child outcomes were coded into four categories for purposes of evaluating the effects of the interest-based interventions: prosocial behavior (e.g., social play/responsiveness, initiations, positive affect), communication (e.g., joint attention, turn-taking, language competence), performance (e.g., task completion, appropriate nonsocial play/engagement, compliance), and undesirable behavior (e.g., negative affect, avoidance, disruptive behavior).

### 2.6. Method of Analysis

Data reported in the studies was used to make either of two types of comparisons: baseline versus interest-based interventions or low (or no) interest-based conditions versus interest-based conditions. All of the baseline versus intervention comparisons included contrasts between intervention and nonintervention conditions. The low-interest versus high-interest intervention comparisons all involved contrasts between different levels or intensity of interests-based practices. In the majority of the studies, the original data were reanalyzed for the purposes of the meta-analysis.

Cohen's *d *effect sizes for the baseline versus intervention [91] and the low-interest versus high-interest [92] comparisons were used to estimate the size of effect for the interest-based interventions. These were calculated as the mean difference between the contrasting conditions divided by the pooled standard deviation for the two conditions. For purposes of the meta-analysis, the effect sizes for the relationships between interest-based interventions and negative or aberrant child behavior outcomes which were expected to yield negative sizes of effect were reversed to reflect the fact that interest-based interventions would be associated with less behavior excesses. The distribution of the effect sizes was first examined to identify outlines. Only 5 of 174 effect sizes were two or more standard deviations above or below the mean. Lipsey and Wilson [93] recommend that outliners be recoded to a value equal to the effect size at two standard deviations above or below the mean to ensure that the outliners are “kept from being so extreme relative to other effect sizes in the distribution [so as to not] greatly distort the analysis” (Page 108).

The average effect sizes for the relationship between the comparative intervention conditions and the study outcomes were used to evaluate the influence of interest-based learning on the child outcomes. The 95% confidence intervals for the average effect sizes were also used for substantive interpretation. The lower and upper bounds of a confidence interval are a measure of the precision of the average effect size estimate [94]. The *Z*-test was used to evaluate the strength of the relationship between the interest-based interventions and the child outcomes. *Z* provides an estimate of the amount of covariation between an independent or predictor variable and study outcomes [12].

## 3. Results

### 3.1. Preliminary Analyses

We first examined the average effect sizes for the relationships between type of interest-based comparison (baseline versus intervention and low interest versus high interest) and the study outcomes to determine if the comparative conditions could be combined or the results needed to be analyzed separately. The average effect size for the baseline versus intervention comparisons was 3.16 (95% CI = 2.49–3.83), *Z* = 9.38, *P* = .0000, and the average effect size for the low- versus high-interest comparisons was 1.50 (95% CI = 1.07–1.94), *Z* = 6.89, *P* = .0000. Inasmuch as the average effect size for the former type of comparisons was twice as large as the latter type of comparison, all further analyses are reported for the two types of comparisons separately.

The reason the average effect size for the low- versus high-interest comparisons was smaller than those for the baseline versus intervention comparisons has to do with the fact that almost all of the low-interest conditions had interest-based features of elements that presumably had some development-instigating characteristics. For example, in those studies comparing adult-selected activities versus child-selected activities, the adults most likely used knowledge of the children's preferences to decide which toys, materials, activities, and so forth were used to affect child behavior [77, 79]. The same was likely the case for other types of low- versus high-interest comparisons (e.g., [63, 65, 67, 72]).

### 3.2. Type of Interest-Based Intervention

**Figure 1:**
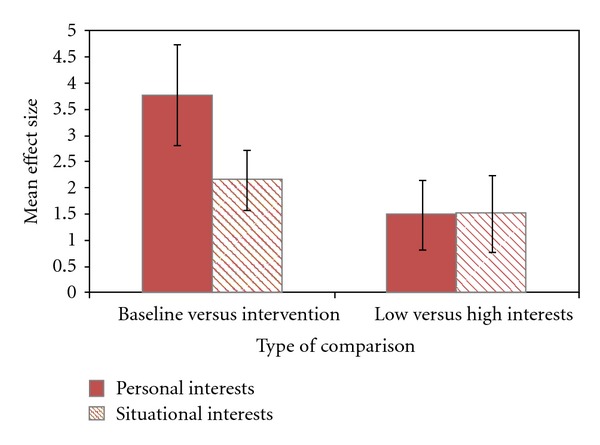
Average effect sizes and 95% confidence intervals for incorporating either personal or situational child interests into the interest-based interventions (*Z*_*s*_ = 4.60  to  7.87, *P* = .0000 for all average effect sizes).

The relationships between the personal and situational interest-based interventions and the child outcomes are shown in Figure 1. Both types of interest-based interventions were associated with positive child outcomes as evidenced by statistically significant *Z*-tests and nearly identical confidence intervals (*Z*_*s*_ = 4.60  to  7.87, *P*_*s*_ = .0000). The influences of personal interest-based interventions, however, were almost twice as large compared to situational interest-based interventions for the baseline versus intervention comparisons. In contrast, both types of interests had similar effects on the study outcomes for the low- versus high-interest comparisons. 

The reasons there are discernable differences for type of interest-based interventions in the baseline versus intervention comparisons but not for the low- versus high-interests-based comparisons are very much the same as that described in the Section 3.1. Whereas the low-interest-based conditions in all likelihood included some interest-based features (e.g., limited but nonetheless some child choice), this was not the case for the baseline versus intervention comparisons. In the latter kind of study, the baseline conditions almost always involved observation or assessment of child behavior in the absence of any child interest, choice, or preference. The results from the baseline versus intervention condition comparisons indicated that incorporating the personal interests of young children with autism spectrum disorders into the interventions proved more effective in terms of changes or improvements in child outcomes compared to engaging children in situationally interesting activities.

### 3.3. Type of Core Feature Intervention

Next we assessed whether targeting one of the three core features of autism spectrum disorders mattered in terms of influencing child outcomes by categorizing the studies in terms of the main or primary focus of the interventions.  Table 1 shows the extent to which interventions targeting the core features of autism spectrum disorders had like or unlike effects on the study outcomes. The interventions, regardless of their focus, were all effective in changing or improving child behavior as evidenced by statistically significant *Z*-tests. The pattern of results, however, showed that interventions focusing on communication or interpersonal behavior were more effective than interventions focusing on restricted and repetitive behavior. The large confidence intervals indicate that the average effect sizes are not precise estimates of the sizes of effects of interventions categorized similarly. Therefore, the characteristics of the core feature interventions therefore most likely differed in terms of some undetermined dimensions.

### 3.4. Type of Child Outcome

The relationship between the interest-based interventions and the four outcome categories described earlier is shown in Table 2. The interest-based interventions were effective in terms of influencing changes or improvements in all four outcome categories as evidenced by statistically significant *Z*-tests. The results indicated that the interest-based interventions were associated with increased or improved child prosocial behavior, child communication competence, and child performance and associated with decreased negative and undesirable child behavior. But again the large confidence intervals indicate that the interventions differentiately influenced the child outcomes for reasons not readily apparent.

Ten of the 24 studies included both positive and negative child outcome measures which permitted a direct test of whether increases in prosocial, communication, and performance outcomes were associated with concomitant decreases in undesirable child behavior [59, 60, 63–65, 70, 71, 73, 77, 78, 80, 83, 84]. The average effect sizes for the baseline versus intervention comparisons were 3.70 (95% CI = 2.56–4.84, *Z* = 6.66, *P* = .0000) for the positive child outcomes and 2.00 (95% CI = 0.29–3.70, *Z* = 2.46, *P* = .0139) for the negative child outcomes. Similarly, the average effect sizes for the low- versus high-interest comparisons were 1.70 (95% CI = 0.95–2.44, *Z* = 4.68, *P* = .0000) for the positive child outcomes and 1.38 (95% CI = 0.81–1.95, *Z* = 5.59, *P* = .0000) for the negative child outcomes. In both sets of analyses, the results showed that interest-based interventions were effective in terms of increasing and improving positive child outcomes while at the same time decreasing aberrant child behavior. 

### 3.5. Moderator Analyses

Whether the relationship between the interest-based interventions and the study outcomes were influenced by nonintervention variables was investigated by moderator analyses [95]. The moderator variables included severity of autism spectrum disorders (mild, moderate, mixed), child age (23–40, 41–60, 61–72 months), and intervention setting (home, clinic, school). The interventions were all effective regardless of the moderators as evidenced by statistically significant *Z*-tests for all within moderator variable groups (*Z*_*s*_ = 2.47  to  7.853, *P*_*s*_ = .01  to  .0000). There were however, differences in the average sizes of effect as a function of the different moderator groups. The interest-based interventions were more effective when used with children with mild impairments compared to children with either moderate or mixed impairments, with older children, and when implemented in the children's homes. 

**Figure 2:**
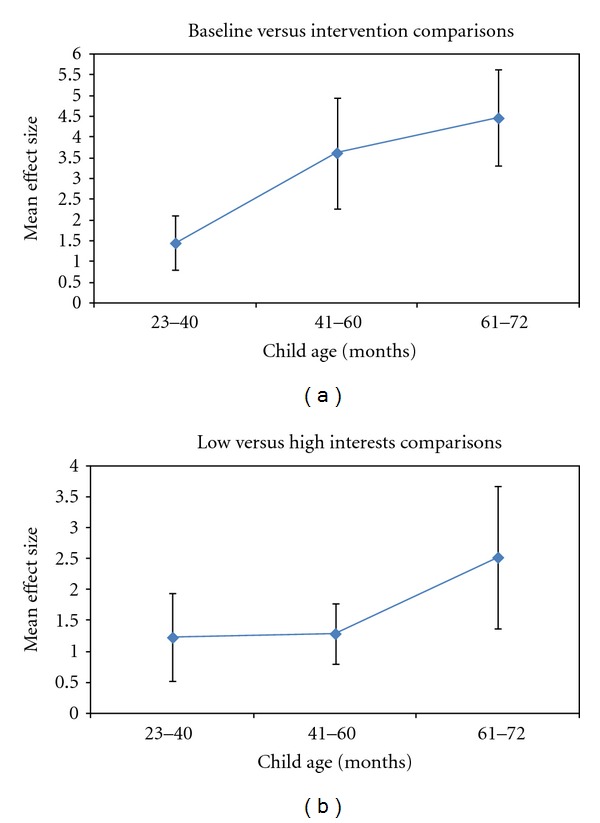
Influences of child age on the relationship between the interest-based interventions and the child outcomes (*Z*_*s*_ = 3.57  to  7.83, *P*_*s*_ = .0004  to  .0000 for all average effect sizes).

An example of a moderator effect is presented for child age. Although all of the average effect sizes for the three child age groups were statistically significant (*Z*_*s*_ = 3.57  to  7.85, *P*_*s*_ = .0004  to  .0000), child age nonetheless moderated the relationship between the interest-based interventions and the child outcomes. The relationship between child age and the average effect sizes is shown in Figure 2. As can be seen, there are discernable upward trends in the relative effectiveness of the interventions for both types of comparative conditions as a function of child age.

Similar types of trends were found for both the child severity and intervention setting moderators. For example, the average sizes for effect for child severity for the baseline versus intervention and low- versus high-interests comparisons were, respectively, 4.51 (95% CI = 3.26–5.76, *Z* = 7.31, *P* = .0000) and 2.19 (95% CI = 1.05–3.33, *Z* = 3.98, *P* = .0001) for mild impairments, 3.36 (95% CI = 2.38–4.34, *Z* = 6.97, *P* = .0000) and 1.14 (95% CI = 0.47–1.81, *Z* = 3.495, *P* = .0005) for moderate impairments, and 0.79 (95% CI = 0.13–1.45, *Z* = 2.47, *P* = .0134) and 1.33 (95% CI = 0.86–1.80, *Z* = 5.85, *P* = .0000) for children having a mix of impairments. The same type of trend was found for intervention setting for both types of comparisons. The average effect sizes for home-based interventions were the largest, followed by clinic-based interventions, and then the classroom interventions. The average effect sizes for the latter were nonetheless statistically significant as evidenced by *Z*_*s*_ = 4.47  and  6.63, *P* = .0000.

## 4. Discussion

Results showed that the different ways in which the interests of young children with autism spectrum disorders were incorporated into early intervention practices promoted child prosocial behavior and decreased child behavior excesses. Results showed as well that interest-based early intervention practices targeting the communication and interpersonal core features of autism spectrum disorders were more effective than interventions targeting restricted or repetitive child behavior. The interest-based interventions were also found to change or improve child behavior in a number of different areas and domains. The findings, taken together, indicate that interest-based early intervention is an effective practice for increasing a variety of prosocial, communication, and performance outcomes and decreasing undesirable behavior [48, 49].

The findings as a whole are best understood by considering the results from the different sets of analyses together. By doing so, one can see that incorporating the personal interests of young children with autism spectrum disorders into communication or interpersonal interventions is more likely to optimally increase prosocial child behavior while at the same time decreasing aberrant child behavior. This pattern of results is very much like findings from studies of young children with other types of developmental disabilities [35, 96, 97] as well as young children without developmental disabilities or delays [98–100]. It therefore seems that the interests of young children with autism spectrum disorders function as a behavioral- and development-instigating characteristic in the same or similar manner as for other young children with and without developmental disabilities or delays.

The findings, although encouraging, need to be placed in proper perspective, especially in terms of the limitations and weaknesses of the original studies and therefore the validity of the meta-analysis results. These include the following: (1) the small number of studies and especially the small number of participants in the studies, (2) differences in the research methodologies and the types of comparisons that could be made as part of the meta-analysis, (3) the limited information in the original research reports in terms of the severity of child impairments and the fact that no studies of young children with either Rett or Asperger's syndrome could be located, and (4) the lack of consistency in how children's interests were defined and incorporated into the interventions. The manner in which the meta-analysis was conducted addressed all of these limitations by the ways in which constructs were operationally defined albeit with somewhat limited success. The results highlight the need for better designed and implemented studies and especially studies that include operationalized measures of child interests.

The types of studies that are needed to advance our understanding of the characteristics and consequences of interest-based interventions with young children with autism spectrum disorders include the following: studies that include *a priori* operationally defined interest measures as well as operationally defined methods and procedures for incorporating interests into early intervention practices, inclusion of children with diagnoses across the entire autism spectrum, children who differ in their severity of disabilities where severity is assessed using both reliable and valid assessment scales, and inclusion of outcome measures that tap both prosocial and aberrant behavior so that the differential consequences of the interventions can be discerned. In addition, larger simple size studies and studies that include children with autism spectrum disorders younger than 3 years of age would permit a determination of interest-based interventions that are in fact warranted with very young children diagnosed with autism spectrum disorders. 

### 4.1. Conclusion

Incorporating the interests of young children with autism spectrum disorders into early intervention practices was found to be a promising practice for promoting prosocial child behavior and decreasing both behavior excesses and undesirable child behavior. The results from the meta-analysis are particularly encouraging in light of the fact that advances in the early identification of infants and toddlers with autism spectrum disorders [15] which makes it possible to intervene early before behavior markers associated with those disorders become firmly established [17]. Interest-based intervention practices constitute one approach to intervening early [48, 49] and therefore may be a practice of choice for affecting the behavior, competence, and development of young children with autism spectrum disorders. Nonetheless, there is a clear need for better designed studies with larger number of study participants to be able to identify the conditions under which interest-based interventions are determined to be an evidence-based practice.

## Figures and Tables

**Table 1:** Average effect sizes and 95% confidence intervals for interventions targeting different core features of autism spectrum disorders.

Type of comparison/core features	Number		Average effect size	95% Confidence interval	*Z*-test
	Studies	Effect sizes			
*Baseline versus intervention*					
Communication	5	21	4.04	2.76–5.33	6.55***
Interpersonal	4	16	3.66	2.40–4.93	6.16***
Repetitive behavior	4	40	1.56	0.54–2.57	3.10*

*Low versus high interest*					
Communication	3	21	1.52	0.55–2.50	3.26**
Interpersonal	3	10	3.78	1.75–5.80	4.23***
Repetitive behavior	5	32	0.85	0.48–1.21	4.75***

*P = .0019, **P = .0011, ***P = .0000.

**Table 2:** Average effect sizes and 95% confidence intervals for the different categories of child outcomes.

Type of comparison/child outcomes	Number		Average effect size	95% Confidence interval	*Z*-test
	Studies	Effect sizes			
*Baseline versus intervention*					
Prosocial behavior	4	10	3.55	1.76–5.33	4.49****
Communication	5	16	5.03	3.61–6.45	7.55****
Performance	8	46	2.92	1.87–3.98	5.59***
Negative behavior	6	25	2.25	0.92–3.56	3.50**

*Low versus high interest*					
Prosocial behavior	7	20	2.53	1.37–3.70	4.56****
Communication	4	8	1.07	0.17–2.20	2.22*
Performance	9	36	1.17	0.56–1.78	3.91***
Negative behavior	7	13	1.11	0.64–1.57	5.23****

*P = .03, **P = .0005, ***P = .0001, ****P = .0000.
